# The Crystal Structure of a Biological Insulated Transmembrane Molecular Wire

**DOI:** 10.1016/j.cell.2020.03.032

**Published:** 2020-04-30

**Authors:** Marcus J. Edwards, Gaye F. White, Julea N. Butt, David J. Richardson, Thomas A. Clarke

**Affiliations:** 1 School of Biological Sciences, University of East Anglia, Norwich NR4 7TJ, UK; 2 School of Chemistry, University of East Anglia, Norwich NR4 7TJ, UK

**Keywords:** extracellular electron transfer, shewanella, porin-cytochrome complex, iron oxidation, metal reduction, respiration, outer membrane protein, electrogenic bacteria, geobacter, decaheme

## Abstract

A growing number of bacteria are recognized to conduct electrons across their cell envelope, and yet molecular details of the mechanisms supporting this process remain unknown. Here, we report the atomic structure of an outer membrane spanning protein complex, MtrAB, that is representative of a protein family known to transport electrons between the interior and exterior environments of phylogenetically and metabolically diverse microorganisms. The structure is revealed as a naturally insulated biomolecular wire possessing a 10-heme cytochrome, MtrA, insulated from the membrane lipidic environment by embedding within a 26 strand β-barrel formed by MtrB. MtrAB forms an intimate connection with an extracellular 10-heme cytochrome, MtrC, which presents its hemes across a large surface area for electrical contact with extracellular redox partners, including transition metals and electrodes.

## Highlights

• The 20 hemes of a 3-component complex are arranged to move electrons across 185 Å
• A β-barrel and 10-heme cytochrome form an insulated transmembrane nanowire
• An extracellular 10-heme cytochrome has a large surface area for electron exchange
• The hemes of both cytochromes are packed with a maximum inter-heme distance of 8 Å

## Graphical Abstract

**Figure:**
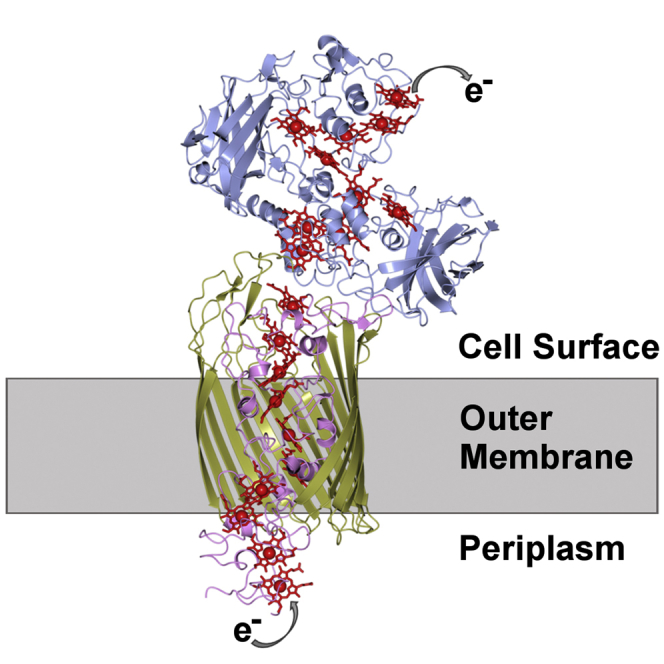

## Teaser

Structural analysis shows how a naturally insulated molecular “wire” conducts electrons across lipid membranes in bacteria while protecting the membrane from redox damage and facilitating extracellular electron exchange

## Introduction

The outer membranes of Gram-negative bacteria are naturally insulative and prevent the indiscriminate exchange of electrons between the cell and environment. However, a number of important bacterial processes require the conductance of electrons across the outer membrane. These include the transfer of intracellularly derived electrons out of the cell to external electron acceptors or the import of electrons into the cell from extracellular electron donors in order to support the formation of reducing equivalents for carbon fixation (Shi et al., 2016). The ability to directly transfer electrons into and out of bacterial cells is also of increasing interest for biotechnological applications including microbial fuel cells, microbial electrosynthesis, unbalanced fermentation, and bio-electronic interfaces (Bursac et al., 2017, Lovley, 2012, Rabaey and Rozendal, 2010). Mechanistically, this process requires electrons to be transported across the outer membrane of the cell in a controlled pathway that prevents detrimental redox side reactions, such as the generation of reactive oxygen species. Thus, an “insulated” electron transfer complex is required.

The Gram-negative *Shewanella* is one of the most studied bacterial genera capable of transferring electrons out of the cell to solid-phase Fe(III) and Mn(IV) minerals in order to support anaerobic respiration (Beblawy et al., 2018, Edwards et al., 2018). We have previously purified an icosaheme protein complex comprising three subunits (MtrA, MtrB, MtrC) from the outer membrane of *Shewanella oneidensis* and shown that this Mtr complex is able to conduct electrons bidirectionally across proteoliposome bilayers sustaining electron transport rates over 8,500 e s^–1^ (White et al., 2013). In liposome studies, the direction of electron transfer is dependent on the relative redox potentials across the membrane. Extraliposomal sodium dithionite is capable of reducing intraliposomal methyl viologen or small tetraheme cytochrome (STC), which can then be used to reduce extravesicular iron oxides and chelates (Edwards et al., 2018, White et al., 2013).

**Figure S1:**
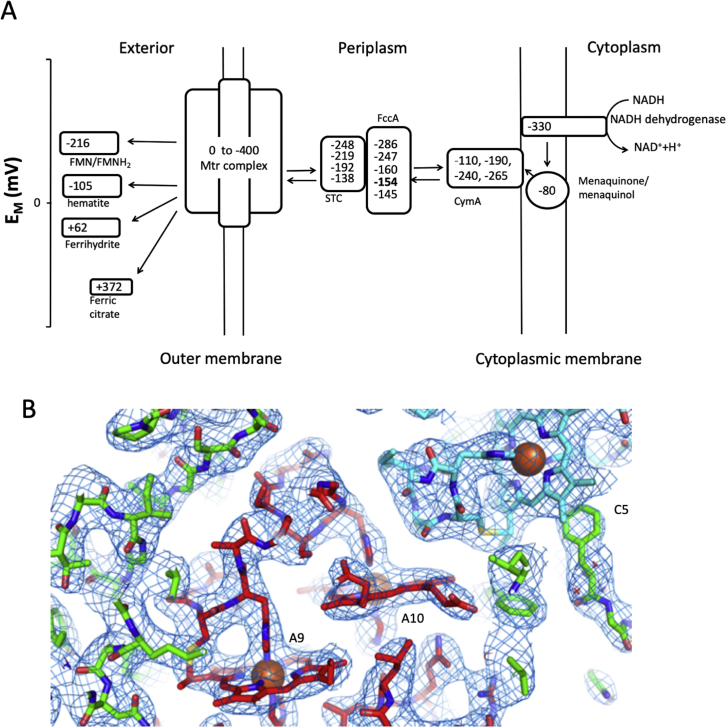
Reduction Potentials and Electron Density Map of the Mtr Complex, Related to Figure 1 (A) Scheme depicting the electron transfer pathway between NADH dehydrogenase and *Shewanella oneidensis* MR-1. The midpoint potentials of cofactors of the different components of the pathways are given versus Standard Hydrogen Electrode at pH 7. The FccA FAD/FADH_2_ potential is highlighted in bold. (Adams et al., 2010, Firer-Sherwood et al., 2008, Harada et al., 2002, Hartshorne et al., 2009, Pessanha et al., 2009, White et al., 2013) (B) 2Fo-Fc electron density map around Heme A9, A10 of MtrA and Heme C5 of MtrC. Stick representation of the Mtr complex. Carbons are colored according to chain with MtrA colored red, MtrB colored green, MtrC colored blue. Nitrogen atoms are colored blue, with oxygens colored red. The 2Fo-Fc electron density map (blue mesh) is contoured at 1.5 σ.

The reduction potentials of the 20 hemes in the *S. oneidensis* Mtr complex span from approximately 0 to −400 mV versus SHE. This allows the complex to accept electrons from the menaquinol dehydrogenase CymA, via the periplasmic cytochromes small tetraheme cytochrome (STC) and periplasmic fumarate reductase (FccA), and transfer them to extracellular soluble and insoluble electron acceptors (Figure S1A). Within this complex, the ten heme MtrC is the cell-surface module required when solid-phase minerals serve as terminal electron acceptors (Coursolle and Gralnick, 2010, Hartshorne et al., 2007). MtrAB is proposed to span the outer membrane and provide electrical connection between the catabolic electron transfer network within the cell, and the electron dispersing MtrC outside the cell (Edwards et al., 2018). Despite these findings, critical information about the complex, including the topology and interactions of all three components, has been missing until now. Here, we present the structure of an Mtr complex at 2.7 Å resolution. This structure reveals MtrAB to be a naturally insulated molecular wire that is able to conduct electrons across the lipidic outer membrane and deliver them to MtrC, which positions its hemes to optimize electron distribution to extracellular redox partners.

## Results and Discussion

### Structure of a 20-Heme Electron Transfer Complex

**Figure 1:**
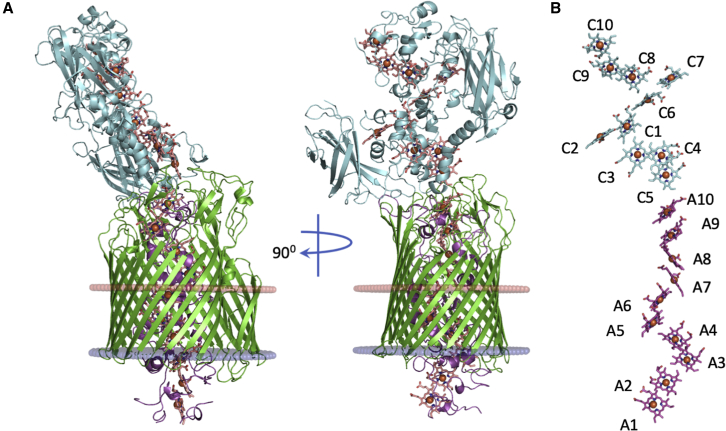
The X-Ray Crystal Structure of the Mtr Complex from *S. baltica* OS185 (A) Cartoon views of the Mtr complex rotated by 90 degrees. The extracellular MtrC (blue) is associated with the surface of the transmembrane porin MtrB (green). MtrA (magenta) is sheathed inside MtrB with the N-terminal protruding from the periplasmic side. The predicted lipid bilayer position of the Mtr complex is shown as red and blue discs representing extracellular and periplasmic faces, respectively (Lomize et al., 2012). (B) Heme network within the Mtr complex. Hemes are numbered according to the position of the heme attachment motif within the amino acid chain and colored blue (MtrC) or magenta (MtrA). See also Figure S1.

**Figure 2:**
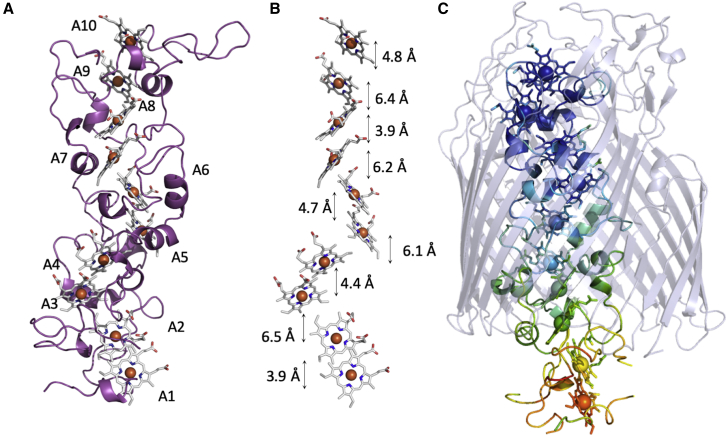
Structural Features of MtrA (A) Cartoon of MtrA with hemes shown as sticks. Hemes are numbered as in Figure 1. (B) Heme arrangement within MtrA. Minimum electron transfer distances between the porphyrin rings of adjacent hemes are shown. (C) Flexibility of MtrA. MtrB is shown in grey in cartoon representation. MtrA is shown in cartoon representation and is coloured with a gradient based upon calculated B factors, from low (∼30 Å^2^) shown in blue to high (∼160 Å^2^) shown in orange. See also Figures S2 and S3.

Despite multiple attempts, the Mtr complex from *S. oneidensis* could not be crystallized. However, homologous Mtr complexes are produced endogenously by different *Shewanella* species (Fredrickson et al., 2008) and screening of several of these provided a single crystallizable hit. The Mtr complex from *Shewanella baltica* OS185 produced crystals that diffracted to a final resolution of 2.7 Å, and data were phased utilizing the anomalous signal from the 20 iron atoms of the c-type hemes (Table 1; Figure S1). The crystal structure reveals a heterotrimeric complex that consists of two cytochromes, MtrC and MtrA, and a beta-barrel protein, MtrB, that sheathes and insulates MtrA (Figure 1A). A network of 20 *bis*-His coordinated hemes spans the complex, forming an electron transfer pathway of 185 Å (Figure 1B). The hemes of MtrA and MtrC are annotated in order of the CxxCH binding motifs to which they are connected, e.g., heme A1 binds to the first motif in the MtrA amino acid sequence and heme C1 binds to the first motif in the MtrC sequence (Figures 1B and 2A).

**Table 1:** Data Collection and Refinement Statistics

Data Collection	MtrC SAD	MtrCAB SAD	MtrCAB Native^a^
Space group	P 2_1_ 2_1_ 2	C 2 2 2_1_	C 2 2 2_1_

**Cell dimensions**			

*a*, *b*, *c* (Å)	90.52, 291.50, 87.20	209.90, 235.16, 98.42	212.04, 234.17, 99.19
α, β, γ (°) (°)	90.00, 90.00, 90.00	90.00, 90.00, 90.00	90.00, 90.00, 90.00
Resolution (Å)	90.52–2.29 (2.35–2.29)	117.58–3.41 (3.50–3.41)	106.02–2.70 (2.81–2.70)
*R*_merge_	0.098 (1.790)	0.143 (0.879)	0.115 (2.194)
*CC*_*1/2*_	1.0 (0.48)	1.0 (0.49)	1.00 (0.73)
*I* / σ*I*	17.0 (1.2)	9.5 (2.2)	9.9 (0.8)
Completeness (%)	99.5 (98.2)	99.5 (99.9)	99.0 (97.5)
Redundancy	12.6 (10.2)	6.4 (6.4)	5.8 (5.4)

**Refinement**			

Resolution (Å)	87.20–2.29		73.25–2.70
No. reflections	104,187		49,734
*R*_work_ / *R*_free_	0.183/0.228		0.224/0.257

**No. atoms**			

Protein	13,424		22,512
Ligand/ion	1,306		1496
Water	914		22

***B* factors**			

Protein	53.00		63.11
Ligand/ion	46.50		54.52
Water	51.77		37.58

**RMSDs**			

Bond lengths (Å)	0.004		0.013
Bond angles (°)	0.780		1.753

Each dataset was collected from a single crystal. Values in parentheses are for highest-resolution shell.

a Statistics for data collection prior to anisotropic correction utilizing STARANISO. Data were truncated along the surface defined by I/σ*(I)* = 1.2. Corrected data were used for subsequent refinement.

### Structure of the Embedded Decaheme Cytochrome MtrA

**Figure S2:**
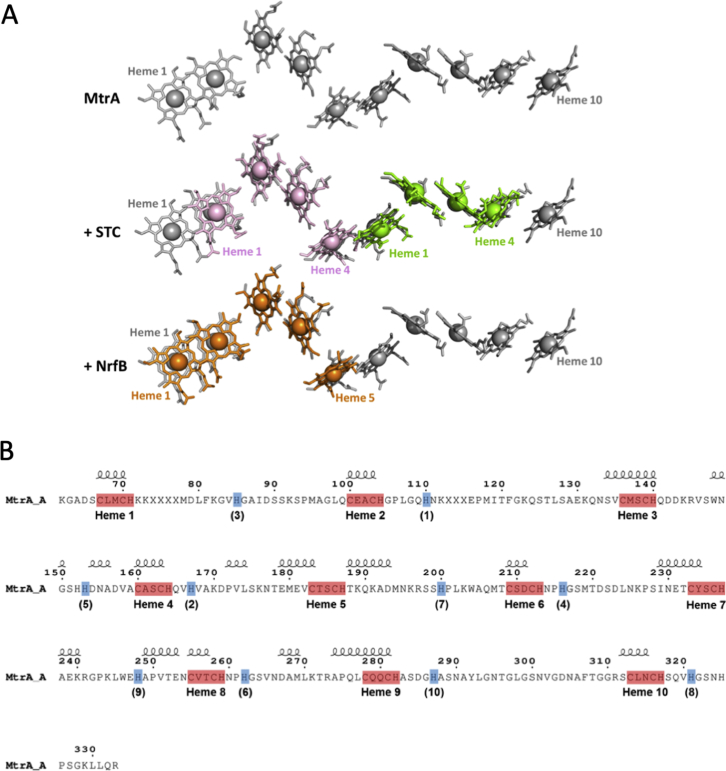
Cofactor Arrangement and Secondary Structure of MtrA, Related to Figure 2 (A) Structural alignment of hemes from *S.baltica* OS185 MtrA (gray) with hemes from two copies of *S.oneidensis* MR1 STC (PDB: 1M1P, pink/green, RMSD: 1.52 Å/1.64 Å) and hemes from a single copy of *Escherichia coli* K-12 NrfB (PDB: 2OZY, orange, RMSD: 1.74 Å). Structural alignments were performed using Superpose (Krissinel and Henrick, 2004). (B) MtrA residues visible in the crystal structure of MtrCAB. Disordered residues are indicated by an X. Heme binding residues (CxxCH) are highlighted in red. Distal histidines are highlighted in blue with corresponding heme number shown in parentheses. Secondary structure elements are represented above the amino acid sequence. Figure was modified from the output of ENDscript 2.0 (Robert and Gouet, 2014).

Hemes A1 to A10 of MtrA are arranged such that neighboring pairs have alternating parallel and perpendicular porphyrin ring planes for which the closest edge-edge distances lie between 3.9 and 6.5 Å (Figure 2B). Similar configurations are found in the heme chains of smaller cytochromes from *Shewanella* and other bacteria. The heme chain of STC (Leys et al., 2002) of *Shewanella sp*. can be superposed over hemes A2–A5 and hemes A6–A9 of MtrA with root-mean-square deviation (RMSD) of 1.52 Å and 1.64 Å, respectively. The pentaheme chain of NrfB from *Escherichia coli* (Clarke et al., 2007) can be superposed over hemes A1–A5 of MtrA with an RMSD of 1.74 Å (Clarke et al., 2007, Leys et al., 2002) (Figure S2A). The sequence identity between STC and MtrA in the aligned regions is only 22%–23%, and the corresponding sequence identity between NrfB and MtrA is only 33%. However, the conservation of the heme arrangement observed in these structures suggests that STC, NrfB, and MtrA might share a common ancestor, with MtrA arising from a gene duplication. The arrangement of MtrA heme A9 and A10 cannot be superposed on hemes from either STC or NrfB, possibly because the orientation of heme A10 has altered to facilitate electron transfer to extracellular MtrC.

The hemes of STC and NrfB are redox active within the same potential window as MtrA, specifically 0 to −400 mV versus SHE (Clarke et al., 2004, Firer-Sherwood et al., 2008), and a maximum electron flux of 3 × 10^6^ s^–1^ though hemes 1–4 of STC has been calculated (Jiang et al., 2017) in agreement with measurements of heme-heme electron transfer rates in that protein (van Wonderen, 2019). Thus, MtrA may support similarly rapid electron transfer across the 80 Å heme chain between heme A1 and heme A10. This distance is more than sufficient to facilitate the collection of electrons originating within the periplasm, transport across the ∼40 Å outer cell membrane and delivery to the extracellular environment.

The MtrA polypeptide has very little secondary structure, with only 20% of the polypeptide chain composed of helices and the remaining 80% consisting of flexible loops (Figure 2A; Figure S2). This is consistent with previous small-angle X-ray scattering (SAXS) analysis of isolated MtrA in solution (Firer-Sherwood et al., 2011). Kratky analysis of these data revealed a peak at low scattering angles, consistent with a folded protein, but increased at higher scattering angles consistent with a flexible MtrA.

**Figure S3:**
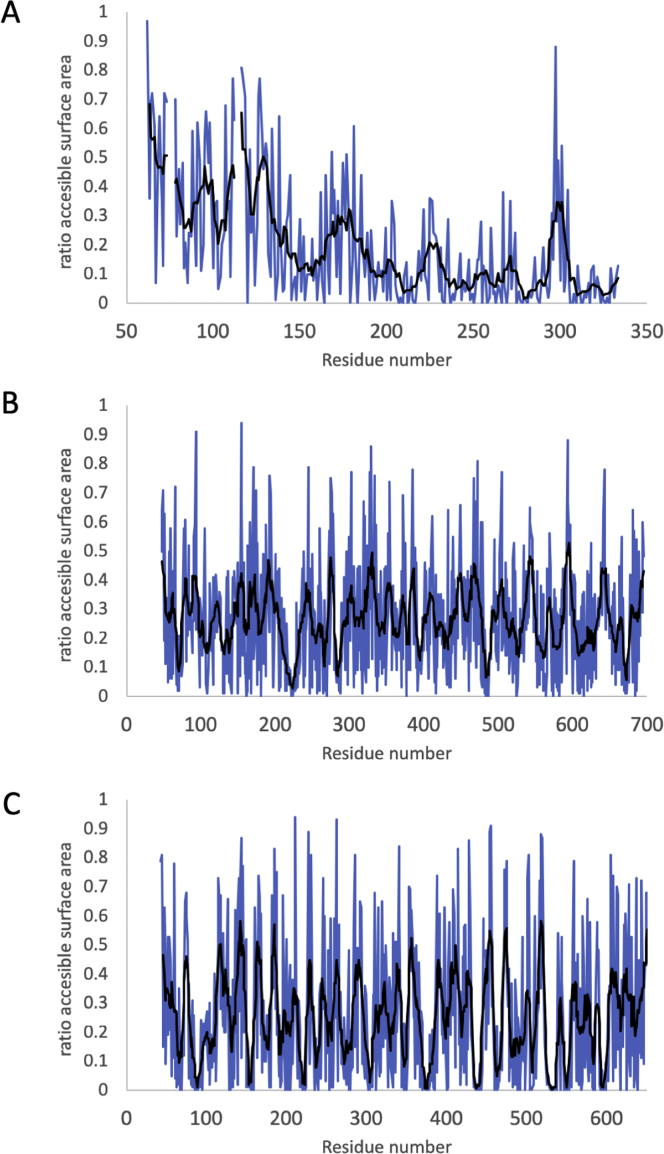
Solvent Accessible Surface Areas of Amino Acids for Each Subunit within the Mtr Complex, Related to Figures 1 and 2 Surface area is calculated using AreaImol (Saff and Kuijlaars, 1997) as the ratio of surface area for each residue compared to a completely exposed equivalent residue. Amino acid plots are shown for (A) MtrA, (B) MtrB and (C) MtrC. Blue line is the accessible surface area for individual residues while the black line is a moving window average of the accessible surface area for 8 residues.

As a component of the Mtr crystal structure, the flexibility of MtrA can be observed through the temperature (B) factors of the peptide backbone, where higher values are associated with increased chain mobility. The B factors of MtrA increase from the externally facing C terminus to the periplasmic facing N terminus (Figure 2C; Figure S3). Loops in the C-terminal half of the MtrA form hydrogen bonds with internally facing charged MtrB side chains, restricting mobility. In contrast, the interactions between the N-terminal half of MtrA and MtrB are much less extensive, which increases the mobility of the periplasmic facing side of MtrA. The MtrA N terminus is the most mobile region and projects out of MtrB into the periplasmic compartment, with heme A1 located approximately 20 Å inside the periplasm. This could facilitate interactions between heme A1 and soluble periplasmic proteins such as STC and fumarate reductase FccA, which have been previously shown to be electron donors to MtrA (Edwards et al., 2018, Sturm et al., 2015).

### Structure of the Transmembrane MtrB Sheath

**Figure 3:**
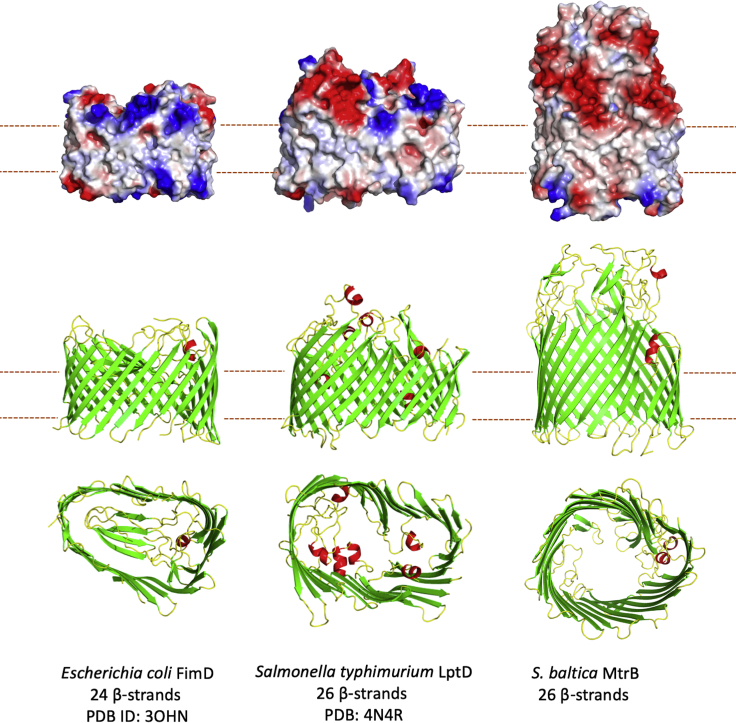
Structure and Comparison of the β-Barrel Proteins FimD, LptD, and MtrB (Top) Electrostatic maps of the surfaces of each β-barrel. (Middle and Bottom) Side and bottom view of each structure shown as cartoons with helices colored red, strands colored green, and loops colored yellow. Electrostatic surfaces generated using APBS in Pymol with potentials scaled from −0.5 V (red, negatively charged) to +0.5 V (blue, positively charged).

Solubility of the Mtr complex in the lipidic outer membrane is conferred by MtrB, a hydrophobic barrel comprised of 26 antiparallel β strands (Figures 1A and 3). The interactions between MtrB and MtrA allow MtrA to be positioned across the outer membrane while insulating the MtrA hemes from the outer membrane environment, preventing non-specific reduction of membrane soluble exogenous molecules such as oxygen, which could result in the generation of reactive oxygen species that in turn lead to cellular damage (e.g., lipid peroxidation). The MtrB porin orients MtrA so the heme chain is perpendicular to the membrane and electron transfer away from the cell is optimized.

**Figure 4:**
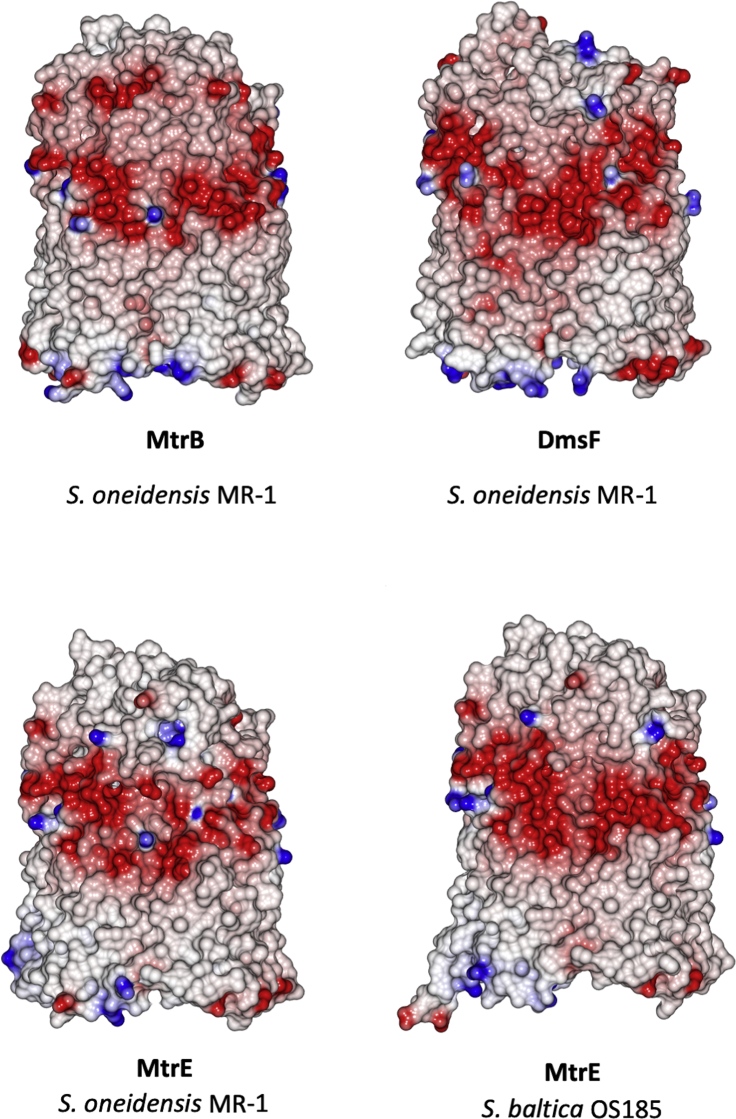
Electrostatic Surface Maps of Molecular Models of MtrB Homologs from *S. oneidensis* and *S. baltica* Homology models of MtrB homologs generated using Phyre2 (Kelley et al., 2015) with *S. baltica OS185* MtrB as a template. Electrostatic surfaces calculated using CCP4MG with potentials scaled from −0.5 V (red, negatively charged) to +0.5 V (blue, positively charged).

MtrB, with overall dimensions of approximately 70 × 55 × 45 Å, consists of tight turns on the periplasmic face and surface loops extending ∼45 Å on the extracellular side of the membrane. The overall structure of MtrB is similar to that of other outer membrane secretion proteins (e.g., Figure 3). For example, MtrB is approximately the same size as the 26-strand lipopolysaccharide transporter LptD, and the 24-strand pilin subunit transporter FimD (Botos et al., 2016, Phan et al., 2011) (Figure 3). The amino acid composition of the extracellular loops gives the MtrB surface a uniform negative charge. Structural modeling of the *Shewanella oneidensis* MR-1 porins MtrE and DmsF reveals that, while the negatively charged extracellular surfaces are conserved in the MtrB protein family (Figure 4), they are not observed in electrostatic surface maps of the aforementioned FimD and LptD β-barrel proteins (Figure 3). Like MtrB, DmsF and MtrE are components of outer membrane electron transport complexes that contain extracellular catalytic domains, in these cases DmsAB and MtrF, respectively (White et al., 2016). It is therefore likely that for MtrB and its homologs these negatively charged residues help in the docking of the extracellular catalytic domains, possibly by preventing the negatively charged *Shewanella* lipopolysaccharide (Korenevsky et al., 2002) from binding to the external surface of the membrane embedded porin:cytochrome *c*omplexes.

**Figure 5:**
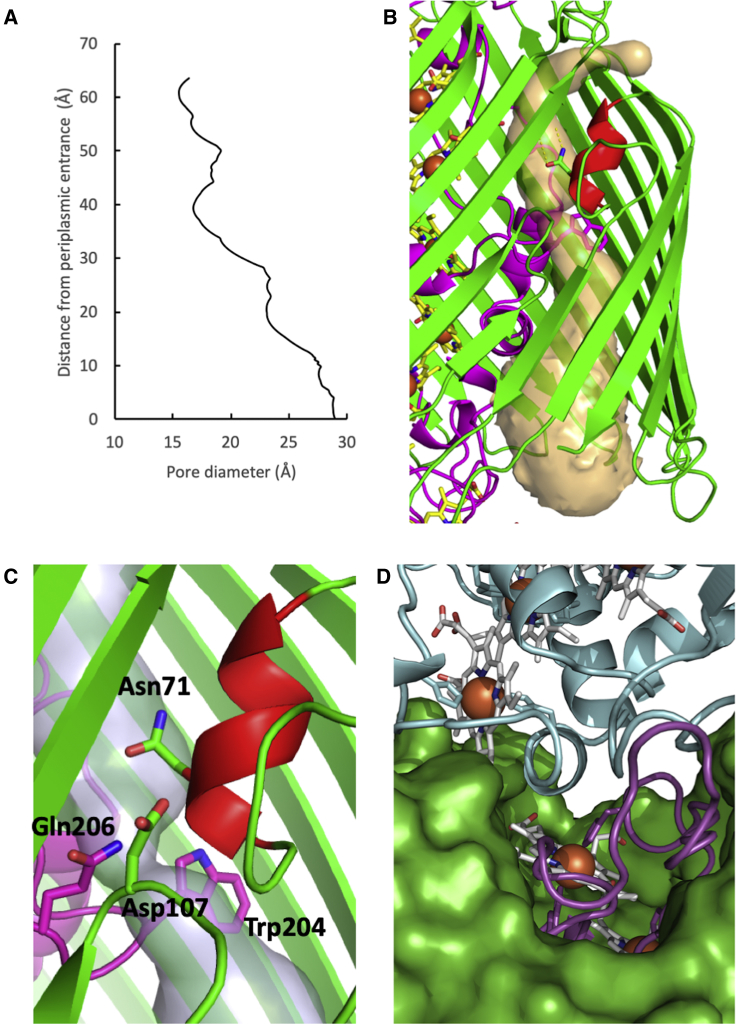
Structural Properties of MtrAB Porin Cytochrome (A) Change in radius of MtrB central cavity from the periplasmic opening to the extracellular surface as measured using CAVER (Pavelka et al., 2016). (B) Cartoon of the MtrAB helix-capped channel. The MtrB channel (semi-transparent yellow) identified using CAVER. (C) Close-up of the helix capped channel in MtrAB, helix is coloured red with Asn_71_, Asp_107_ of MtrB and Trp_2__04_, Gln_206_ shown as sticks. (D) The interface between MtrA (magenta), MtrC (blue), and MtrB (green) with hemes A10, C5, and histidine ligands shown as sticks. See also Figures S3 and S4.

The aperture of the pore defined by MtrB is wider at the periplasmic face (∼30 Å) than at the cell exterior (∼15 Å) where access to the outside is restricted by the folded surface loops (Figures 3 and 5A). The pore is of sufficient size to allow folded MtrA (∼80 × 30 × 35 Å) to insert into the periplasmic opening of MtrB but prevents MtrA from escaping to the cell exterior, effectively trapping it inside MtrB. Thus, assembly of the full Mtr complex is dependent on “stalled” excretion of MtrA by MtrB, and association with MtrC that is translocated across the outer membrane by the type 2 secretion system (Shi et al., 2008).

At the N terminus of MtrB, the first four β strands are shorter than the following 22 strands. Predicted to lie within the lipid bilayer, these shorter strands do not interact with MtrA and define a small solvent channel that runs between MtrA and MtrB (Figure 5B). The diameter of the periplasmic facing side of the channel is ∼5 Å, sufficient to allow free diffusion of water in and out of the periplasmic facing side of MtrB. The channel is capped at the extracellular surface of the outer membrane by a small α helix formed by the MtrB surface loop between β strands 1 and 2 (Figure 5C). This helix is stabilized by several hydrogen bonds, including two between Asn_71_ and the backbone of Tyr_133_ on β strand 5. The conserved Trp_204_ of MtrA is positioned underneath the cap and causes a bottleneck that restricts diffusion of charged and polar molecules from the extracellular face of the channel. However, there are polar residues, Asn_71_ of MtrB, and Gln_206_ of MtrA, that could stabilize water molecules on either side of the channel and a charged residue Asp_107_ that could participate in proton exchange. The role of this channel is unclear, but it may allow for proton transport that has been suggested to occur through the Mtr complex during anaerobic respiration (Okamoto et al., 2017).

### The MtrC Domain Is Oriented to Maximize Distribution of Electrons to Terminal Acceptors

**Figure S4:**
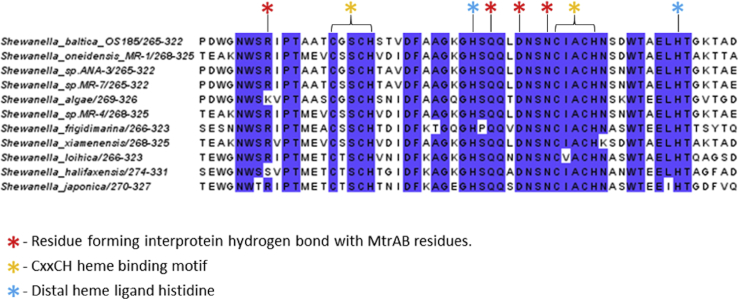
Sequence Alignment of Amino Acids of MtrC, which Form an Interface with MtrA Based upon the Structure of MtrCAB from *Shewanella baltica* OS185, Related to Figure 5 MtrC sequences were aligned with Clustal Omega (Sievers and Higgins, 2018)and formatted with Jalview (Waterhouse et al., 2009).

At the cell surface, the surface loops of MtrB largely cover MtrA so that only MtrA residues 284 to 306 and heme A10 are presented for interaction with MtrC (Figure 5D). Interprotein electron transfer from heme A10 is facilitated by the positioning of MtrC heme C5 within an edge-edge distance of 8 Å. The amino acid sequence around heme C5 is highly conserved within the MtrC clade of outer membrane cytochromes (Figure S4). This conserved sequence contains residues that form hydrogen bonds with MtrAB, allowing association of MtrC to the surface of MtrAB. In the absence of MtrC, the exposed edge of the heme A10 porphyrin indicates that reduction of extracellular substrates by MtrAB should be possible and is consistent with previous studies that showed MtrC knockout mutants of *S. oneidensis* were capable of reduction of soluble Fe(III) chelates but not of insoluble iron oxides (Coursolle and Gralnick, 2010).

**Figure S5:**
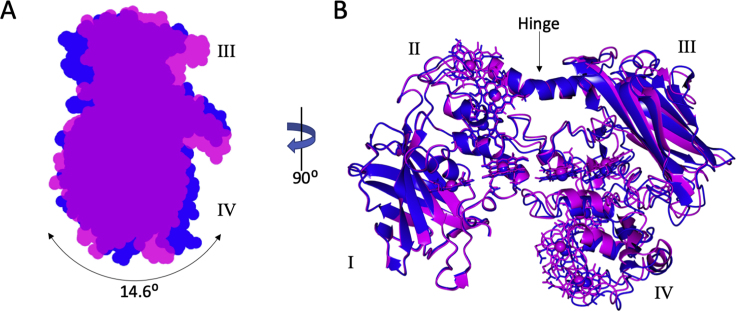
Hinge Movement in MtrC, Related to Figures 1 and 5 (A) The 3 copies of MtrC_sol_ in the asymmetric unit display domain I/II movement relative to domains III/IV with a maximal rotation of 15 degrees. The domain movement between the two extremes of the crystal structure are shown in magenta and blue. The rotation was measured using DYNDOM (Hayward and Lee, 2002). (B) Residues 289-300 located within the alpha-helix linking domains II and III formed a hinge region. Domain movement also resulted in changes in relative orientation of hemes C1 and C6 with less than a 1 Å change in the edge-to-edge distance of the heme porphyrin rings.

To aid initial model building for the Mtr complex, an X-ray crystal structure was obtained for a soluble form of *S. baltica* OS185 MtrC (MtrC_sol_). The structure of the monomeric MtrC_sol_ was similar to that of MtrC from *S. oneidensis* MR-1 described previously (Edwards et al., 2015). MtrC_sol_ consists of 4 domains: two split beta-barrel domains, domains I and III, and two α-helical domains, domains II and IV. These domains serve as a scaffold for 10 bis-His coordinated hemes arranged in a “staggered cross” formation (Edwards et al., 2015). The three MtrC_sol_ monomers within the asymmetric unit of the crystal displayed domain I/II movements relative to domains III/IV. These domain movements centered round a hinge region formed by residues 289–300 located within the alpha helix linking domains II and III. The location of the hinge point suggests this range of motion would not be restricted in the Mtr complex, giving conformational flexibility to MtrC. Analysis by DynDom (Hayward and Lee, 2002) showed a maximal rotation of 15 degrees (Figure S5A) and that changes in relative orientation of hemes C1 and C6 were accompanied by <1 Å change in the edge-to-edge distance of the corresponding porphyrin rings (Figure S5B).

**Figure S6:**
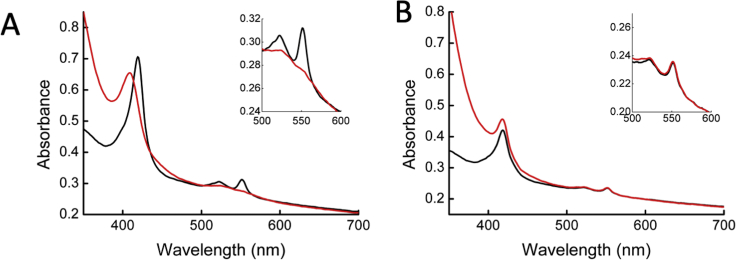
UV-Visible Spectrum of *S. oneidensis* MR-1 and *S. oneidensis* Δ*MtrCAB-omcA-DEF* Cells before and after Addition of 0.5 mM Fe(III) Citrate, Related to Figures 1, 2, and 5 Spectra of cells were recorded using a UV-visible spectrophotometer equipped with an integrating sphere to minimize light scatter from cells. The spectrum of *S. oneidensis* cells incubated anaerobically in the presence of 50 mM formate showed spectral features consistent with reduced c-type cytochromes, which are assembled within the periplasm (A, black line). After addition of Fe(III)citrate to the cells, the spectrum changes consistent with the hemes within the cells being fully oxidised in the presence of an electron acceptor (A, red line). The spectrum of *S. oneidensis* strain lacking the *mtrCAB* gene cluster did not change before (B, black line) or after addition of Fe(III)citrate (B, red line).

In the Mtr complex, MtrC is angled on the surface of MtrAB so that the more insulated side of MtrC faces the membrane surface, while the negatively charged heme propionates face toward the environment thereby providing a suitable surface for direct electron transfer to large extracellular substrates (Figure 1). Heme C10 is presented to the environment ∼90 Å above the hydrophobic bilayer core and is located close to the PTPTD amino acid sequence previously identified as a possible hematite binding hydroxylated motif (Lower et al., 2008); therefore, this may be a primary route for direct electron transfer to insoluble substrates. Previously, modeling of the electronic micro-environments of MtrC suggested that the net driving force between hemes C5 and C10 is rather small, allowing for bi-directional electron transfer through the heme chain formed by C5, C4, C3, C1, C6, C8, C9, and C10 (Figure 1B) (Barrozo et al., 2018). However, the redox potentials of hemes C2 and C7 are higher than the other hemes, raising the possibility that these hemes serve as both capacitors and junctions in an Mtr electrical circuit (Jiang et al., 2019). The arrangement and orientation of MtrC on the surface therefore allows electrons to flow in from a single heme, C5, and then be distributed across the surface of MtrC, with multiple hemes acting as potential electron donor sites, thereby allowing electron transfer to both insoluble and soluble electron acceptors. This arrangement of hemes within the Mtr complex also implies that electron transport through the outer membrane would not be rate limiting during respiration. To support this, the redox state of the cytochrome pool in living *S. oneidensis* MR-1 cells was explored using UV-visible spectroscopy to measure the spectrum of the cytochromes expressed in the periplasm and on the cell surface. Addition of an extracellular acceptor, Fe(III)citrate, immediately oxidized the cytochrome pool of *S. oneidensis* MR-1. However, the cytochrome pool of *S. oneidensis* mutant lacking the *mtr* cluster remained reduced, demonstrating the role of the Mtr complex as a gateway to the electrons that accumulate in the cytochromes of the *S. oneidensis* MR-1 periplasm (Figure S6).

### Wider Implications of the Mtr Structure

The crystal structure of the Mtr complex reveals how Gram-negative bacteria have evolved insulated molecular wires for the movement of electrons across the outer membrane of the cell. This evolution appears to have involved harnessing electron transferring cytochromes and outer membrane secretory proteins. This core MtrAB electron conduit reveals how extracellular electron transport, which is also widespread in both photoautotrophic and chemoautotrophic iron-oxidizing bacteria, can be accomplished efficiently.

Recently, the structure of OmcS, an extracellular cytochrome from *Geobacter sulferreducens* PCA was resolved (Wang et al., 2019). OmcS was revealed as a hexaheme cytochrome that assembled into a polymeric cable containing a contiguous linear heme chain extending from the cell surface. The contrast between the structures of OmcS and the surface exposed MtrC highlight the different mechanisms used to transfer electrons to extracellular substrates, with *Shewanella* MtrC dispersing electrons through a trifurcated heme arrangement, and *Geobacter* OmcS through a linear heme chain with a single heme terminus. We note that a porin:cytochrome complex functionally similar to Mtr has been identified in *G. sulferreducens* PCA *(*Liu et al., 2014*)*. Thus, it is possible that this complex transports electrons across the outer membrane of *Geobacter* and delivers them to the OmcS polymer for subsequent long-range transport through the extracellular matrix.

Previous studies indicate the likely rate-limiting step of electron transfer across a membrane by the Mtr complex is electron transfer to/from the complex, rather than inter-heme electron exchange (Richardson et al., 2012, White et al., 2016). The measured rate of 8,500 *e* s^–1^ for Mtr-mediated electron transfer in proteoliposomes from internalized reduced methyl viologen to extraliposomic ferrihydrite represents a lower limit for electron flux through the complex (White et al., 2016) but is far greater than the ∼1 *e* MtrC^–1^ s^–1^ required to support anaerobic respiration (Ross et al., 2009). The true rate of electron transfer from MtrA heme A1 to MtrC heme C10 is difficult to quantify, due to the similar spectroscopic signatures of the 20 bis-histidine coordinated hemes within the large Mtr complex. Computational analysis of MtrC by molecular simulation has suggested electron transfer rates of ≈10^5^ s^–1^ between heme C5 and heme C10 (Jiang et al., 2019). Assuming the closely arranged hemes of MtrA support similar electron transfer rates, then the likely maximum rate of electron transfer across the Mtr complex is likely to be ∼10–100 times greater than those observed to date and will likely require careful simulation and experimentation to confirm.

## STAR★Methods

### Key Resources Table

**Table:** 

REAGENT or RESOURCE	SOURCE	IDENTIFIER
**Bacterial and Virus Strains**		

*Shewanella baltica* OS185	Dr Liang Shi, Department of Biological Sciences and Technology, School of Environmental Studies, China University of Geosciences. China	N/A
*Shewanella oneidensis* MR-1	Dr Liang Shi, Department of Biological Sciences and Technology, School of Environmental Studies, China University of Geosciences. China	N/A
*Shewanella oneidensis* MR-1 Δ*mtrB*-*mtrD*	Dr Liang Shi, Department of Biological Sciences and Technology, School of Environmental Studies, China University of Geosciences. China	LS527
*Shewanella oneidensis* MR-1 Δ*mtrB*-*mtrD* pMJE001	This manuscript	ME001

**Chemicals, Peptides, and Recombinant Proteins**		

N,N-Dimethyldodecylamine N-oxide (LDAO)	Merck	Cat#40234
Trimethylamine N-oxide (TMAO)	Merck	Cat#92277
L-Lauroylsarcosine	Merck	Cat#61739
Triton X-100	Merck	Cat#X100
Polyethylene glycol 6000	Merck	Cat#81260
Polyethylene glycol 8000	Merck	Cat#1546605
Polyethylene glycol 10000	Merck	Cat#8.21881
Polyethylene glycol monomethyl ether 2000	Merck	Cat#202509
2-Methyl-2,4-pentanediol (MPD)	Merck	Cat#112100

**Deposited Data**		

Structure of *Shewanella baltica* OS185 MtrC_sol_	This manuscript	PDB: 6QYC
Structure of *Shewanella baltica* OS185 Mtr complex	This manuscript	PDB: 6R2Q

**Recombinant DNA**		

pBAD-MtrCsol (*S.baltica* OS185)	This manuscript	pMJE001

**Software and Algorithms**		

CRANK2	Skubák and Pannu, 2013	http://www.ccp4.ac.uk
COOT	Emsley et al., 2010	https://www2.mrc-lmb.cam.ac.uk/personal/pemsley/coot/
REFMAC	lNicolls, 2012.	https://www.ccp4.ac.uk
STARANISO	Tickle et al., 2018	http://staraniso.globalphasing.org
PHENIX	Adams et al., 2010	http://www.phenix-online.org/
AutoProc	Vonrhein, 2011	https://www.globalphasing.com/autoproc/
CCP4MG	McNicholas et al., 2011	http://www.ccp4.ac.uk/MG/
Pymol	Schrödinger	https://pymol.org/2/

**Other**		

U-4100 Spectrophotometer with Integrating Sphere	Hitachi	N/A
Vivaspin 20 10 kDa MWCO centrifugal concentrator	Sartorius	Cat#VS2001
Vivaspin 20 100 kDa MWCO centrifugal concentrator	Sartorius	Cat#VS2041
Vivaflow 200 30 kDa MWCO casette	Sartorius	Cat#VF20P2
HiLoad 26/600 Superdex 200 pg	G E Healthcare	Cat#28989336
Micro DispoDialyzer, 50 kDa MWCO Regenerated Cellulose Membrane	Harvard Apparatus	Cat#74-0720
Q Sepharose Fast Flow Chromatography Resin	G E Healthcare	Cat#17051001
MRC 2 Lens Crystallization Microplate	SWISSCI	MRC96T-UVP
DEAE Fast Flow Chromatography Resin	G E Healthcare	Cat#17070901

### Resource Availability

#### Lead Contact

Further information and requests for resources and reagents should be directed to and will be fulfilled by the lead contact Tom Clarke (tom.clarke@uea.ac.uk).

#### Materials Availability

All unique/stable reagents generated in this study are available from the Lead Contact with a completed Materials Transfer Agreement.

#### Data and Code Availability

The accession numbers for the coordinates of *S. baltica* Mtr complex and MtrC reported in this paper are RCSB PDB: 6R2Q and 6QYC respectively.

### Experimental Model and Subject Details

*Shewanella baltica* OS185, *Shewanella oneidensis* MR-1 (ATCC 700550) and *Shewanella oneidensis* MR-1LS527 (Δ*mtrB-mtrD*, locus tags SO_1776-SO_1782) were obtained from our laboratory culture collection.

To obtain biomass for *Shewanella baltica* OS185, 20 l of LB media supplemented with 20 mM Fe(III)citrate, 50 mM sodium lactate pH 7.8 were inoculated with 2 mL of *S. baltica* OS185, grown aerobically overnight in LB, per liter of media and incubated for 24 hours at 30°C, shaking at 180 rpm. Cells were harvested by centrifugation at 6000 g at 4°C for 20 minutes and resuspended in 20 mM HEPES pH 7.8 in final volume of ∼350 ml.

A soluble *S. baltica* MtrC construct, MtrC_sol_, was synthesized (Eurofins genomics) with codon-optimization for expression in *Shewanella oneidensis* MR-1. The soluble construct was generated by replacing the N-terminal signal peptide and lipid-anchor attachment site of MtrC (*S.baltica* OS185) (residues 1-26) with the N-terminal signal peptide of MtrB from *S. oneidensis* MR-1 (residues 1-24). The MtrC_sol_ construct was then cloned into a pBAD-202D TOPO vector, utilizing the topo cloning site while introducing a stop codon and a ribosome binding site before the start codon of the MtrC_sol_ gene, in order to prevent fusion to a his-patch thiredoxin sequence present in the vector, to give vector pMJE001. Plasmid pMJE001 was transformed into *S.oneidensis* LS527 by electroporation to give ME001.

### Method Details

#### Purification of the *S. baltica* Mtr complex

*S. baltica* OS185 cells were lysed by two passages through a French Pressure system at 16,000 psi. Cell debris was removed by centrifugation at 5000 g for 20 minutes at 4°C and bacterial cell membranes were isolated by centrifugation at 185,000 *g* at 4°C for 2 hours. Membranes were washed by resuspending in 20 mM HEPES pH 7.8 to a final volume of ∼300 mL and stirred at 4°C overnight. L-lauroylsarcosine was added to a final concentration of 2% w/v and stirred for 1 hour at 4°C to preferentially solubilise the inner membrane. The soluble fraction was separated by centrifugation at 185,000 *g*, 4°C for 2 hours and discarded. The pelleted outer membranes were then solubilised by resuspending in 20 mM HEPES pH 7.8, 5% Triton X-100 (v/v) to a volume of ∼300 mL and stirred overnight at 4°C.

The solublised membranes were then centrifuged at 185,000 *g*, 4°C for 2 hr. The soluble fraction was loaded onto a 150 mL Q-Sepharose column pre-equilibrated with 20 mM HEPES pH 7.8, 2% Triton X-100 (v/v) (buffer A). The column was washed with 2 column volumes of buffer A. Protein was eluted with a gradient of 0 - 0.5 M NaCl in buffer A over 850 mL collecting 11 mL fractions. Heme containing fractions were analyzed by SDS-PAGE and fractions containing the Mtr complex were pooled and diluted 3-fold with buffer A before being loaded onto a 10 mL Q-Sepharose column pre-equilibrated with buffer A. The column was washed with 10 column volumes of 20 mM HEPES pH 7.8, 5 mM LDAO. The protein was then eluted with 20 mM HEPES pH 7.8, 5 mM LDAO, 0.5 M NaCl.

Protein was concentrated to ∼15 mL using a 100 kDa molecular weight cutoff centrifugal concentrator (vivaspin). The concentrated protein was applied to a Superdex S200 26/600 size-exclusion column pre-equilibrated with 20 mM HEPES pH 7.8, 150 mM NaCl, 5 mM LDAO. 2 mL fractions were collected across the elution peaks and analyzed by SDS-PAGE. Pure MtrCAB fractions from each size-exclusion column run were pooled and concentrated to ∼18 mg/ml. The protein was dialysed against 500 mL of 20 mM HEPES pH 7.8, 100 mM NaCl + 5 mM LDAO overnight at 4°C using 50 kDa molecular weight cutoff dispo-dialysers (Havard Apparatus). Protein was aliquoted into ∼200 μl aliquots and snap frozen in liquid nitrogen before storing at −80°C.

#### Purification of *S. baltica* OS185 MtrC_sol_

4 × 1 l of Terrific Broth media containing 30 μg/ml kanamycin were inoculated with 2 mL / liter of an overnight culture of ME001 and incubated at 30°C, shaking at 180 rpm, to an OD_600_ ∼0.5. L-arabinose was added to a final concentration of 2 mM and cultures were incubated overnight 30°C, shaking at 180 rpm. Growth cultures were centrifuged at 6000 x g to remove cells and the remaining media was concentrated to ∼400 mL utilizing a Vivaflow 200 concentrator with a 30 kDa molecular weight cut-off membrane. The concentrate was dialysed against 5 l of 20 mM HEPES pH 7.8 overnight at 4°C before replacing the buffer with another 5 l of the same buffer for a further 24 hours. The dialysed protein was loaded onto a ∼300 mL DEAE column pre-equilibrated with 20 mM HEPES pH 7.8. The column was washed with 20 mM HEPES pH 7.8 until a stable baseline was obtained and the protein was eluted with a 0-0.5 M NaCl gradient over 850 ml, collecting 11 mL fractions.

Heme containing fractions were analyzed by SDS-PAGE and MtrC containing fractions were pooled and concentrated using a 30 kDa molecular weight cut off spin concentrator (Vivaspin, Sartorius) to ∼20 ml. The concentrate was split into 4 equal 5 mL aliquots and purified by size exclusion chromatography utilizing a Superdex 200 26/600 column equilibrated with 20 mM HEPES pH 7.8 + 150 mM NaCl. Fractions were analyzed by SDS-PAGE and fractions containing highly pure MtrC were pooled and concentrated to ∼20 mg/ml utilizing a 10 kDa molecular weight cut off centrifugal concentrator (Vivaspin, Sartorius).

#### Structure determination of *S. baltica* MtrC_sol_

Crystals of MtrC were obtained from a sitting-drop vapor diffusion setup with 0.4 M sodium acetate pH 4.5 + 5% PEG 6000 + 5% PEG 8000 + 5% PEG 10000 as the reservoir solution. Crystals formed in both 1:1 and 2:1 (reservoir: protein) drops with a total drop volume of 0.6 μl. Crystals were cryo-protected by briefly transferring to a solution of 0.4 M sodium acetate pH 4.5 + 5% PEG 6000 + 5% PEG 8000 + 5% PEG 10000 + 25% ethylene glycol before being vitrified by plunging into liquid nitrogen.

Data were collected at beamline I04 at Diamond Light Source (Table 1). Crystals were of spacegroup P2_1_2_1_2 with typical cell dimensions of a = 90.52 Å b = 291.50 Å c = 87.20 Å. Data were collected at the Fe k-edge, utilizing a wavelength of 1.72 Å, to a final resolution of 2.3 Å. The CRANK2 pipeline was utilized to locate 30 iron atoms, corresponding to the 3 copies of MtrC in the asymmetric unit, provide initial phases and build a partial model (Skubák and Pannu, 2013). A final model was generated through alternating rounds of manual model building with COOT and refinement with REFMAC (Emsley et al., 2010, Winn et al., 2003). Final model was refined to an Rcryst (Rfree) value of 0.183(0.228) and has no residues in the disallowed region of the Ramachandran plot (Table 1). Coordinates have been deposited in the RCSB Protein Data Bank under accession code 6QYC.

#### Structure determination of *S. baltica* MtrCAB

Crystals of the Mtr complex of S. *baltica* OS185 were obtained from two distinct conditions, with the second condition only being obtained from cross-seeding using a seed-stock derived from the first condition. Mtr complex seed-stock crystals were obtained from a sitting-drop vapor diffusion setup in SWISSCI 96-well 2-drop MRC crystallization plates with 50 μl 0.1 M glycine pH 8.5 + 0.3 M trimethanolamine oxide + 12% PEG 2000 MME as the reservoir solution. Crystals formed in both 1:1 and 2:1 (reservoir: protein) drops with a total drop volume of 0.6 μl. These crystals diffracted to ∼5.5 Å resolution and were of spacegroup P321 with typical cell dimensions of a = 210.52 Å, b = 252.21 Å, c = 209.99 Å. Crystals from each drop were crushed and resuspended in their corresponding reservoir solution (∼40 μl) in order to prepare the seed stock. Optimized crystals of the Mtr complex were obtained from a sitting-drop vapor diffusion setup in Swissci 96-well 2-drop MRC crystallization plates with 50 μl 0.1 M bis-tris pH 5.0 + 0.01 M LDAO + 0.4 M CaCl_2_ + 40% MPD as the reservoir solution. Crystals were obtained with both a 0.3:0.2:0.1 and a 0.2:0.3:0.1 protein: reservoir: seed ratio with a final drop volume of 1.2 μl. Crystals did not require further cryo-protection and were vitrified by plunging into liquid nitrogen.

Data were collected at beamline I03 at Diamond Light Source (Table 1). Crystals were of spacegroup C222_1_ with typical cell dimensions of a = 201.150 Å, b = 236.895 Å, c = 98.922 Å. Data were collected at a wavelength of 1.74Å (Fe k-edge) to a resolution of 3.41 Å. 20 Fe-atoms corresponding to the 20 c-type hemes of a single Mtr complex were located using the CRANK2 pipeline which produced phased electron density maps of sufficient quality to build an initial model (Skubák and Pannu, 2013). Native data were collected at a wavelength of 0.98 Å to a final resolution of 2.70 Å resolution. Anisotropy correction of the raw dataset was performed using STARANISO (Vonrhein et al., 2018) with a surface threshold of 1.2/σ(*I*), implemented through the autoPROC pipeline. Anisotropy corrected data were used for model-building and refinement. A complete model of the Mtr complex was built through alternating rounds of manual model building into refined electron density maps with COOT and refinement with Phenix (Adams et al., 2010) (Figure S1).

The final model was refined to an R_*cryst*_ (R_*free*_) value of 0.224 (0.257) and has 12 residues (< 0.8%) in the disallowed region of the Ramachandran plot (Table 1). Coordinates have been deposited in the RCSB Protein Data Bank under accession code 6R2Q. Structures and maps in figures were rendered with either PyMOL (The PyMOL Molecular Graphics System, v.2.0, Schrödinger) or CCP4MG (McNicholas et al., 2011).

#### Spectrophotometric analysis

Shewanella Basal Medium with fumarate as an electron acceptor (SBM+). For 1 l NH_4_Cl (0.46 g), K_2_HPO_4_ (0.225 g), KH_2_PO_4_ (0.225 g), MgSO_4_ (0.117 g) were dissolved in 500 mL water. 23.83 g HEPES, 6.40 g sodium fumarate and 1.2 mL lactic acid were added and water added to give 1 l final volume. The pH of the media was adjusted to pH 7.2 with NaOH and sterilized by autoclaving prior to the addition of 5 ml/l of filter sterilized casamino acids (0.1 g/ml), 5 ml/l of filter sterilized mineral mix and 5 ml/l filter sterilized vitamin mix.

To prepare 1 l of mineral mix, Nitrilotriacetic acid (1.5 g), MnCl_2_ (0.1 g), FeSO_4_ (0.3 g), CoCl_2_ (0.17 g), ZnCl_2_ (0.1 g), CuSO_4_ (0.04 g), AlK(SO_4_)_2_ (0.005 g), Na_2_MoO_4_ (0.09 g), NiCl_2_ (0.12 g), NaWO_4_ (0.02 g), Na_2_SeO_4_ (0.1 g), H_3_BO_3_ (0.005 g) were dissolved in 1 l of water and filter sterilized.

To prepare 1 l of vitamin mix, Biotin (0.002 g), Folic acid (0.002 g), Pyridoxine HCl (0.02 g), Thiamine (0.005 g), Nicotinic acid (0.005 g), Pantothenic acid (0.005 g), Vitamin B12 (0.0001 g), p-aminobenzoic acid (0.005 g), Thiotic acid (0.005 g) was dissolved in 1 l of water and filter sterilized.

10 mL aliquots of SBM+ were transferred to 25 mL sterile containers followed by addition of 100 μL of culture containing *S. oneidensis* MR-1 or *S. oneidensis* LS527 (Δ*mtrB-mtrD*, locus tags SO_1776-SO_1782) grown overnight in LB under aerobic conditions. The *Shewanella* in SBM+ cultures were incubated at 28°C with shaking at 150 rpm overnight. 3 mL aliquots from this culture were transferred to 50 mL sterile containers and sufficient fresh SBM+ added to completely fill the tubes. These were closed with an airtight seal and incubated at 28°C overnight without shaking. The anaerobic cultures were centrifuged at 5,000 rpm for 30 mins and the closed tubes transferred to an anaerobic glove box where all the following steps were performed. The supernatant was decanted, and the cell pellets suspended in anaerobic 20 mM HEPES pH 7.6 buffer. The cell suspensions were centrifuged and suspended in anaerobic 20 mM HEPES pH 7.6 buffer twice more before aliquots were transferred to 1 cm pathlength quartz cuvettes. Anaerobic Fe(III)citrate was injected into the cells to 0.5 mM final concentration using a Hamilton syringe. In order to minimize the effects of background scatter from the cell suspensions, absorbance scans were performed in anaerobically sealed cuvettes using a Hitachi U-4100 spectrophotometer fitted with an integrating sphere.

### Quantification and Statistical Analysis

Quantification and statistical analyses employed in this publication pertain to the analysis and determination of structures by X-ray crystallographic data, which are integral parts of existing algorithms and software used.
